# Different distribution of neuromedin S and its mRNA in the rat brain: NMS peptide is present not only in the hypothalamus as the mRNA, but also in the brainstem

**DOI:** 10.3389/fendo.2012.00152

**Published:** 2012-12-03

**Authors:** Miwa Mori, Kenji Mori, Takanori Ida, Takahiro Sato, Masayasu Kojima, Mikiya Miyazato, Kenji Kangawa

**Affiliations:** ^1^Department of Biochemistry, National Cerebral and Cardiovascular Center Research InstituteOsaka, Japan; ^2^Interdisciplinary Research Organization, University of MiyazakiMiyazaki, Japan; ^3^Molecular Genetics, Institute of Life Sciences, Kurume UniversityFukuoka, Japan

**Keywords:** neuromedin S, neuropeptide, radioimmunoassay, peptide distribution, brain, brainstem

## Abstract

Neuromedin S (NMS) is a neuropeptide identified as another endogenous ligand for two orphan G protein-coupled receptors, FM-3/GPR66 and FM-4/TGR-1, which have also been identified as types 1 and 2 receptors for neuromedin U structurally related to NMS. Although expression of NMS mRNA is found mainly in the brain, spleen, and testis, the distribution of its peptide has not yet been investigated. Using a newly prepared antiserum, we developed a highly sensitive radioimmunoassay for rat NMS. NMS peptide was clearly detected in the rat brain at a concentration of 68.3 ± 3.4 fmol/g wet weight, but it was hardly detected in the spleen and testis. A high content of NMS peptide was found in the hypothalamus, midbrain, and pons–medulla oblongata, whereas abundant expression of NMS mRNA was detected only in the hypothalamus. These differing distributions of the mRNA and peptide suggest that nerve fibers originating from hypothalamic NMS neurons project into the midbrain, pons, or medulla oblongata. In addition, abundant expression of type 2 receptor mRNA was detected not only in the hypothalamus, but also in the midbrain and pons–medulla oblongata. These results suggest novel, unknown physiological roles of NMS within the brainstem.

## INTRODUCTION

Neuromedin S (NMS) is a neuropeptide originally isolated by our group from the rat brain as an additional endogenous ligand for neuromedin U (NMU) receptors types 1 (NMUR1) and 2 (NMUR2), which were previously identified as the orphan G protein-coupled receptors FM-3/GPR66 and FM-4/TGR-1, respectively (Mori et al., 2005). In rats, NMS and NMU are structurally related peptides composed respectively of 36 and 23 amino-acid residues (Brighton et al., 2004; Mori et al., 2005). They have an identical C-terminal amidated seven-residue sequence that is essential for their agonist activity. NMS and NMU precursors are encoded by separate genes mapped to human chromosomes 2q11.2 and 4q12, respectively (Mori et al., 2005). Because these genes are under different transcriptional control, the mRNAs of NMS and NMU have different patterns of distribution (Brighton et al., 2004; Mori et al., 2005), indicating that they have different physiological roles despite having the same agonist effect on their receptors. Expression of NMS mRNA is observed mainly in the brain, spleen, and testis, and the highest level of expression is found in the hypothalamus (Mori et al., 2005). In the rat brain, NMS mRNA is expressed predominantly in the suprachiasmatic nucleus (SCN) of the hypothalamus, although low-level expression of NMS mRNA is also found in other hypothalamic nuclei, such as the arcuate nucleus (ARC), paraventricular nucleus (PVN), and supraoptic nucleus (SON; Mori et al., 2005). In contrast to the mRNA distribution, the distribution of the NMS peptide in the brain has not yet been elucidated.

Because of the high level expression of NMS mRNA in the hypothalamus, there has been a focus on the role of NMS in the brain. The functions of NMS in the brain have been validated by intracerebroventricular (ICV) administration of the peptide to rats: ICV-administered NMS causes a phase shift in circadian rhythm (Mori et al., 2005), suppresses food intake (Ida et al., 2005), reduces urine volume (Sakamoto et al., 2007), increases milk secretion (Sakamoto et al., 2008), and elevates plasma levels of luteinizing hormone (Vigo et al., 2007). The activity of NMS is approximately 10 times as great as that of NMU in the brain, although ICV-administered NMU induces the same responses (Nakahara et al., 2004; Ida et al., 2005; Mori et al., 2005; Sakamoto et al., 2007, 2008). These findings suggest that NMS plays important roles in the brain. However, it is not certain whether these actions described above are the physiological roles of NMS. Interesting results have been obtained by ICV administration of anti-NMS antibody to rats to neutralize the activity of endogenous NMS. In contrast to the actions induced by NMS administration, ICV pretreatment with anti-NMS IgG, but not anti-NMU IgG, blocks the suppression of food intake induced by both ICV and intraperitoneally administered leptin in rats (Nakahara et al., 2010), and ICV administration of anti-NMS antiserum suppresses suckling-induced milk ejection in lactating rats (Sakamoto et al., 2008), suggesting that endogenous NMS plays physiological roles in the regulation of food intake and milk ejection. However, there is no anatomical evidence supporting these roles in the brain: NMS mRNA expression levels are low in the ARC, PVN, and SON in the hypothalamus (Mori et al., 2005), even though these nuclei are involved in the control of feeding behavior and release of oxytocin, a hormone important for milk ejection. Therefore, to understand completely the physiological role of NMS, it is necessary to elucidate the neural circuits formed by NMS-producing neurons in the brain.

Here, to clarify the physiological significance of NMS, we analyzed the regional distribution of NMS in the rat brain at the peptide level. We established a highly sensitive radioimmunoassay (RIA) specific for rat NMS by using a newly prepared antiserum. We then characterized NMS-immunoreactive molecules separated by reversed-phase high performance liquid chromatography (RP-HPLC) and determined the NMS content of the brain and other tissues. An examination of both NMS content and NMS mRNA expression in the various regions of the brain suggested that NMS neurons project directly from the hypothalamus to the brainstem.

## MATERIALS AND METHODS

### PREPARATION OF ANTI-RAT NMS ANTISERA

[Cys^21^]-rat NMS[1-20], a C-terminally Cys-extended rat NMS fragment (positions 1–20), was synthesized and then conjugated to maleimide-activated mariculture keyhole limpet hemocyanin. Anti-rat-NMS serum (batch OB1321-1) was obtained from a Japanese white rabbit after 10 weekly subcutaneous injections of conjugate with adjuvant; a second batch (OB1321-2) was obtained from another rabbit in the same way. The titers of the antisera were determined by RIA.

### RADIOIODINATION OF TRACER LIGAND

A tracer ligand, [Tyr^21^]-rat NMS[1-20], was synthesized and then radioiodinated by using the lactoperoxidase method (Washimine et al., 1994). After radioiodination, monoiodinated tracer was purified by RP-HPLC on a COSMOSIL 5C_18_-AR-II column (4.6 × 150 mm; Nacalai Tesque, Japan). The purified tracer was stored at -30^°^C after the addition of bovine serum albumin at a concentration of 0.1%.

### RIA FOR RAT NMS

The RIA for rat NMS was performed by using the method used for ghrelin, with minor modifications (Hosoda et al., 2000). Each RIA incubation mixture was composed of 100 μl of standard rat NMS or unknown sample in RIA buffer (50 mM Na_2_HPO_4_, 80 mM NaCl, 25 mM EDTA, 0.5% Triton X-100, 0.5% bovine serum albumin, 0.05% NaN_3_, pH 7.4) and 100 μl antiserum diluted with RIA buffer containing 3.1% dextran T40. OB1321-2 anti-rat-NMS antiserum was used at a final dilution of 1/295,000. After a 24-h incubation, 18,000 cpm of [^125^I-Tyr^21^]-rat NMS[1-20] in 100 μl of RIA buffer containing 3.1% dextran T40 was added. Bound and free tracers were separated by precipitation with polyethylene glycol (PEG). After an additional 24-h incubation, 100 μl of 1% γ-globulin in precipitation buffer [50 mM phosphate buffer (pH 7.4) containing 80 mM NaCl and 0.05% NaN_3_] and 500 μl of 16% PEG-6000 in precipitation buffer were added. The solution was then mixed vigorously and incubated for 15 min. The bound tracer was precipitated by centrifugation at 1500 *g* for 20 min, and then the free tracer was removed by aspiration. All procedures described above were performed at 4^°^C. The radioactivity in the precipitation was counted with a γ-counter ARC-600 (Aloka, Japan).

### PREPARATION OF TISSUE EXTRACTS FOR RIA

All procedures using animal experiments were performed in accordance with the Japanese Physiological Society’s guidelines for animal care and the National Cerebral and Cardiovascular Center Research Institute Guide for the Care and Use of Experimental Animals. Ten-week-old male Wistar rats were maintained under 12 h light/dark cycles (light on from 7:00 to 19:00). Food and water were provided *ad libitum* except during the fasting experiments. The brains were sampled during the light period at ZT6 (Zeitgeber time, ZT; ZT0 is lights on and ZT12 is lights off) from free-fed and 48 h-fasted rats, and during the dark period at ZT18 from free-fed rats. Immediately after decapitation of rats, the brain, spleen, and testis were resected. The brain was dissected into the cerebral cortex, cerebellum, hippocampus–thalamus–striatum, hypothalamus, midbrain, and pons–medulla oblongata by using the method of Glowinski and Iversen (1966). After being weighed, each tissue was minced and then boiled for 5 min in 5× volumes of water. After being cooled, acetic acid was added to make a final concentration of 1 M, and then the boiled tissue was homogenized with a Polytron homogenizer (Kinematica, Switzerland). The supernatant of the extract, obtained after a 10-min centrifugation for at 14,000 *g*, was concentrated by evaporation and then subjected to precipitation with acetone at a concentration of 66% acetone. After removal of precipitates, the supernatant was evaporated to remove the acetone, and then loaded onto a Sep-Pak C18 cartridge (Waters, USA) equilibrated with 0.1% trifluoroacetic acid (TFA). After the cartridge had been washed with 10% acetonitrile (CH_3_CN) in 0.1% TFA, the absorbed material was eluted with 60% CH_3_CN in 0.1% TFA, lyophilized, and dissolved in RIA buffer. Portions of samples, equivalent to 20–300 mg wet weight, were subjected to RIA.

The cerebrospinal fluid was collected from three anesthetized 12-week-old male Wistar rats, and then subjected to Sep-Pak purification.

### CHROMATOGRAPHIC CHARACTERIZATION OF IMMUNOREACTIVE NMS IN RAT BRAINS

Immediately after peptide extraction, a portion of sample equivalent to 1.2 g wet weight of whole brain was separated by RP-HPLC on a Symmetry 300 C18 column (3.9 × 150 mm; Waters, USA), with a linear gradient from 10 to 60% of CH_3_CN in 0.1% TFA for 40 min at a flow rate 1 ml/min. The purified cerebrospinal fluid was also subjected to RP-HPLC under the same conditions. The eluates were collected every minute, evaporated, and then lyophilized. The portions of eluate equivalent to 0.5 g wet weight of tissue and 130 μl of cerebrospinal fluid were subjected to RIA for rat NMS. Synthetic rat NMS was also applied to the same RP-HPLC system to compare the retention time with that of immunoreactive (ir)-NMS.

### QUANTITATIVE REVERSE TRANSCRIPTION–POLYMERASE CHAIN REACTION

The brains were sampled from 10-week-old male Wistar rats and various brain regions were dissected as described above. RNA was isolated by using TRIzol reagent (Ambion, USA). Reverse transcription was performed with a QuantiTect reverse transcription kit (Qiagen, USA). Quantitative PCR was conducted with a LightCycler system (Roche, USA) using a SYBR Premix Ex Taq kit (TaKaRa, Japan). The primer set used for rat NMS was 5′-CTCATCTGTGGTCTGCAAAGAG-3′ and 5′-GCATACAGAAGCAGTAGATGAC-3′, and for rat NMUR1 was 5′-CTCAGTCCACTCTATGTACC-3′ and 5′-AGAAGAGCAGTATGGTCGTC-3′, and for rat NMUR2 was 5′-ACTTGAACAGCACAGAGGAG-3′ and 5′-TTCAAAGTCTGATGTCGGAC-3′. Known amounts of rat NMS, NMUR1, and NMUR2 cDNA were used to obtain a standard curve. The data were normalized against total RNA quantity, wet weight of tissues, or 36B4 level.

## RESULTS

### RIA FOR RAT NMS

**FIGURE 1:**
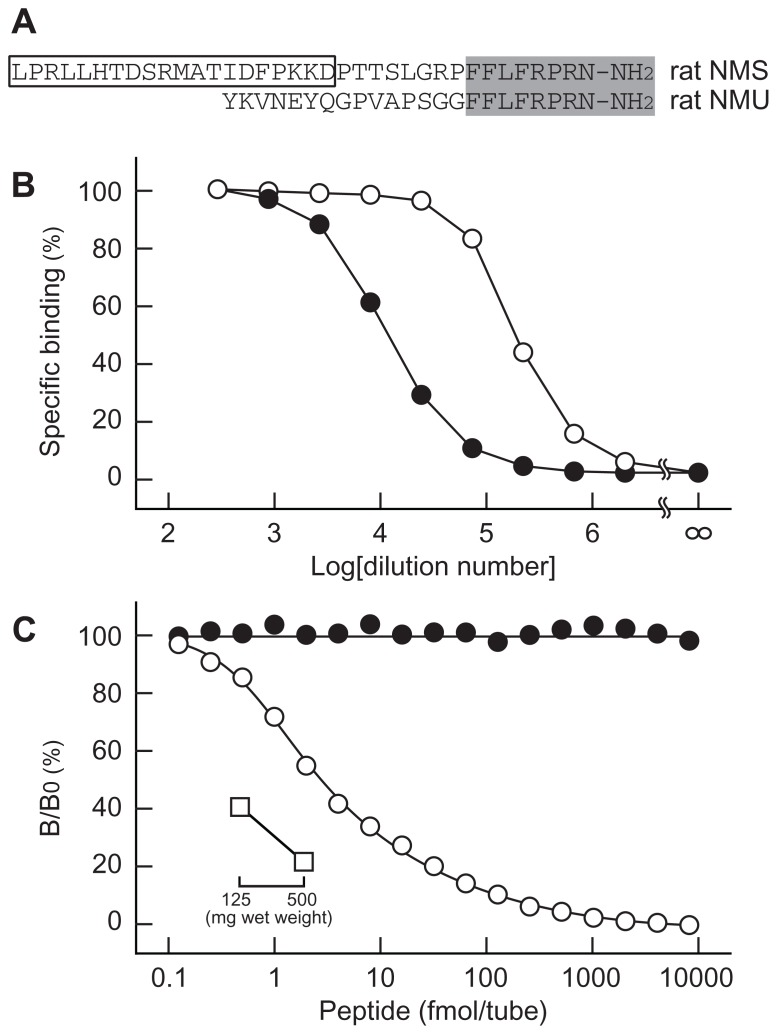
**Characterization of antisera for rat neuromedin S (NMS)**. **(A)** Structural comparison of rat NMS and rat neuromedin U (NMU). Structure conserved in the two peptides is shaded. The box indicates the sequence used as antigen peptide to obtain antisera specific for rat NMS. **(B)** Specific binding curves of antisera. [^125^I-Tyr^21^]-rat NMS[1-20] was incubated with serially diluted OB1321-1 (closed circles) and OB1321-2 (open circles) antisera, and then co-precipitated with antibody in a concentration-dependent manner. **(C)** Standard curve of radioimmunoassay for rat NMS using antiserum OB1321-2. [^125^I-Tyr^21^]-rat NMS[1-20] binding to antibody was displaced by increasing concentrations of rat NMS (open circles) but not rat NMU (closed circles). B/B0 means tracer bound/tracer bound in zero standards. Inset shows the inhibition of tracer ligand binding to antibody by diluted rat brain extract (open squares).

To generate specific antibodies, the N-terminal fragment (positions 1–20) of rat NMS was used as antigen, because this region has no sequence homology to other known peptides including rat NMU, a structurally related peptide (**Figure 1A**). Two types of antisera were obtained; antiserum OB1321-2 exhibited a titer against [^125^I-Tyr^21^]-rat NMS[1-20] approximately 17 times higher than OB1321-1 (**Figure 1B**). Therefore, the OB1321-2 antiserum was used in the subsequent experiments. On the standard RIA curve, the half-maximum inhibition of [^125^I-Tyr^21^]-rat NMS[1-20] binding to antibody by rat NMS was 6.2 fmol/tube, and the peptide was detectable at a low level of 0.25 fmol/tube (**Figure 1C**). The intra- and inter-assay variations were 3.1 and 3.8%, respectively. This antiserum had no cross-reactivity with rat NMU (**Figure 1C**) as well as neuropeptide Y, somatostatin, pituitary adenylate cyclase activating polypeptide, vasoactive intestinal polypeptide, calcitonin gene-related peptide, adrenocorticotropic hormone, α-melanocyte-stimulating hormone, and orexin-A, which are abundantly present in the hypothalamus (data not shown).

In rats, apparent expression of NMS mRNA is found in the brain, spleen, and testis (Mori et al., 2005). Therefore, we quantified NMS peptide in these tissues. The dilution curve generated from whole-brain extract paralleled the standard curve (**Figure 1C**), and the concentration of NMS in rat whole brain was 68.3 ± 3.4 fmol/g wet weight (mean ± SEM, *n* = 3). A small amount of ir-NMS was detected in the cerebrospinal fluid of rats. However, its concentration was not determined, because the value was outside of the range of the standard curve. On the other hand, specific ir-NMS was hard to detect in the spleen and testis (data not shown).

### CHROMATOGRAPHIC CHARACTERIZATION OF ir-NMS IN RAT WHOLE BRAIN

**FIGURE 2:**
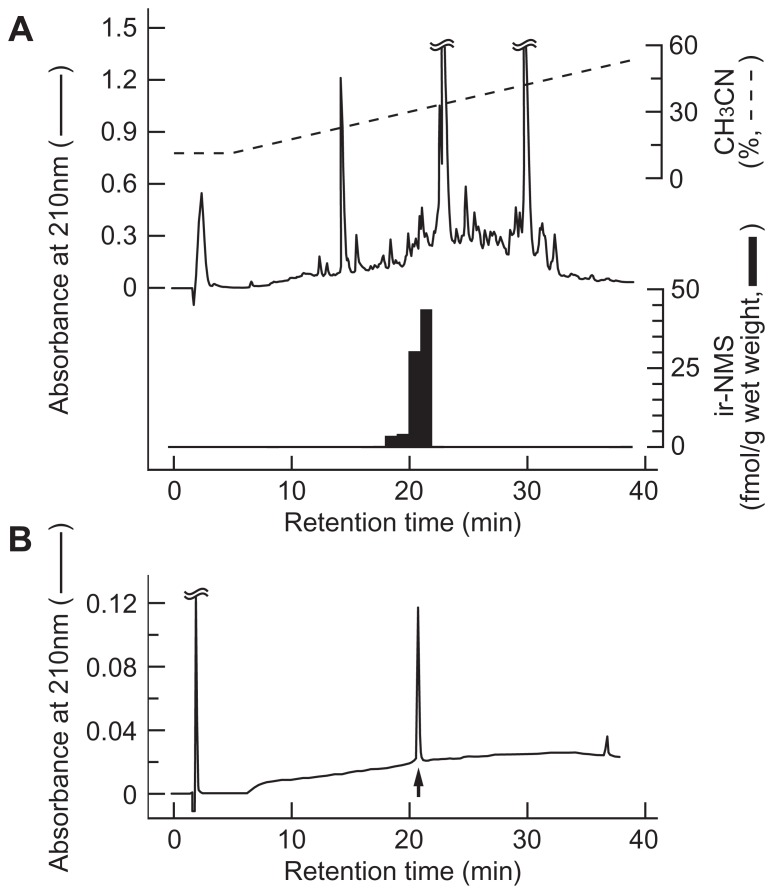
**Representative reversed-phase high performance liquid chromatography (RP-HPLC) profile of neuromedin S (NMS) immunoreactivity in rat brain**. Peptide extract (equivalent to 1.2 g wet weight) from rat whole brain **(A)** and synthetic rat NMS **(B)** were applied to RP-HPLC under the same conditions. Black bars indicate immunoreactive NMS detected by radioimmunoassay **(A)**. Elution position of rat NMS is indicated by the arrow **(B)**.

The molecular forms of ir-NMS found in the extract of rat whole brain were characterized by RIA coupled with RP-HPLC. More than 90% of ir-NMS was eluted as a single peak, with a retention time (20–21 min) identical to that of synthetic rat NMS (**Figure 2**). A very minor ir-NMS was also detected at a retention time of 18–20 min; this corresponded to that observed for oxidized NMS (data not shown). Non-specific immunoreactivity was not observed in any other eluted fractions.

On the other hand, ir-NMS in the cerebrospinal fluid was also eluted at a retention time identical to that of synthetic rat NMS (data not shown).

### REGIONAL DISTRIBUTION OF ir-NMS AND NMS mRNA IN RAT BRAIN

**FIGURE 3:**
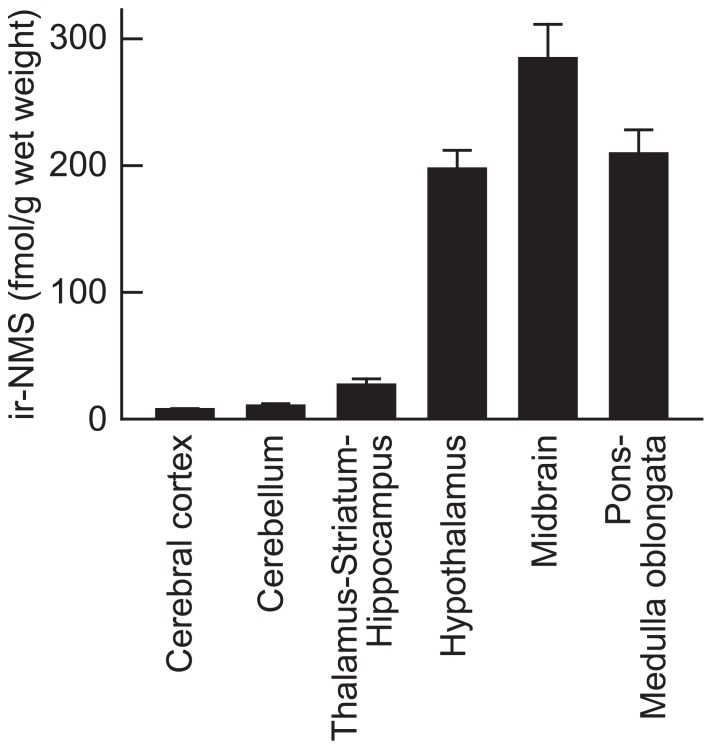
**Regional distribution of immunoreactive neuromedin S (ir-NMS) in rat brain**. Concentrations of ir-NMS in various brain areas were quantified by radioimmunoassay. The brains were sampled during the light period. Data represent means ± SEM of three rats.

The rat brain was dissected into six parts, and both the NMS contents and the NMS mRNA expression levels in these regions were then determined. The highest concentration of ir-NMS was observed in the midbrain (286.5 ± 26.6 fmol/g wet weight, mean ± SEM), followed by the pons–medulla oblongata (208.8 ± 18.7 fmol/g wet weight) and hypothalamus (196.2 ± 15.2 fmol/g wet weight; **Figure 3**). Non-specific immunoreactivity was not found in these brain regions by RIA coupled with RP-HPLC (data not shown). Low levels of ir-NMS were detected in the other brain regions.

**FigurE 4:**
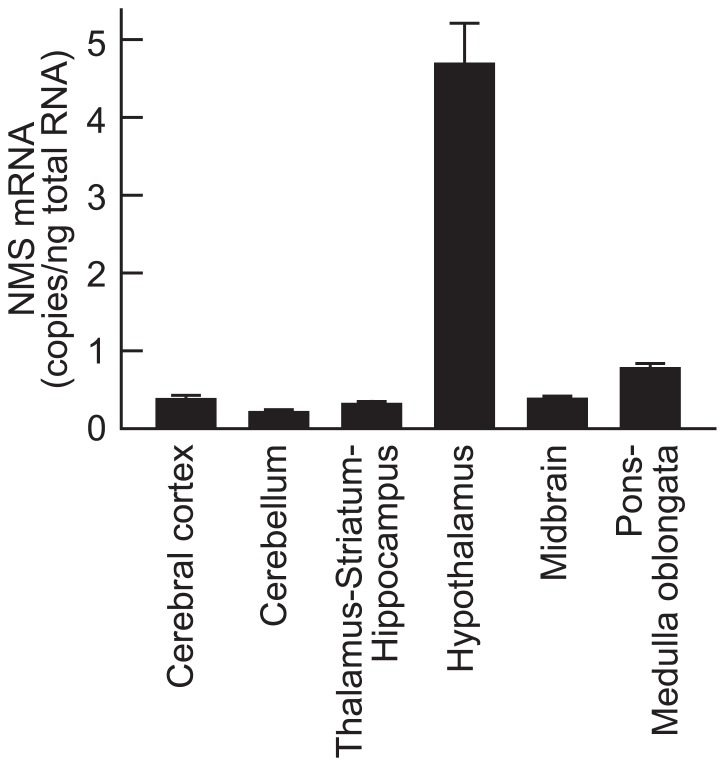
**Expression of neuromedin S (NMS) mRNA in rat brain**. Rat NMS mRNA was quantified by quantitative reverse transcription–polymerase chain reaction analysis. The brains were sampled during the light period. Data represent means ± SEM of three rats.

Abundant expression of NMS mRNA was found only in the hypothalamus (4.69 ± 0.52 copies/ng total RNA, mean ± SEM); low levels of mRNA expression were observed in the other regions (**Figure 4**). Normalization of the mRNA levels against the wet weight of tissues or 36B4 expression did not change this mRNA expression profile in the rat brain (data not shown).

### REGIONAL DISTRIBUTION OF NMUR1 AND NMUR2 mRNA IN RAT BRAIN

**FIGURE 5:**
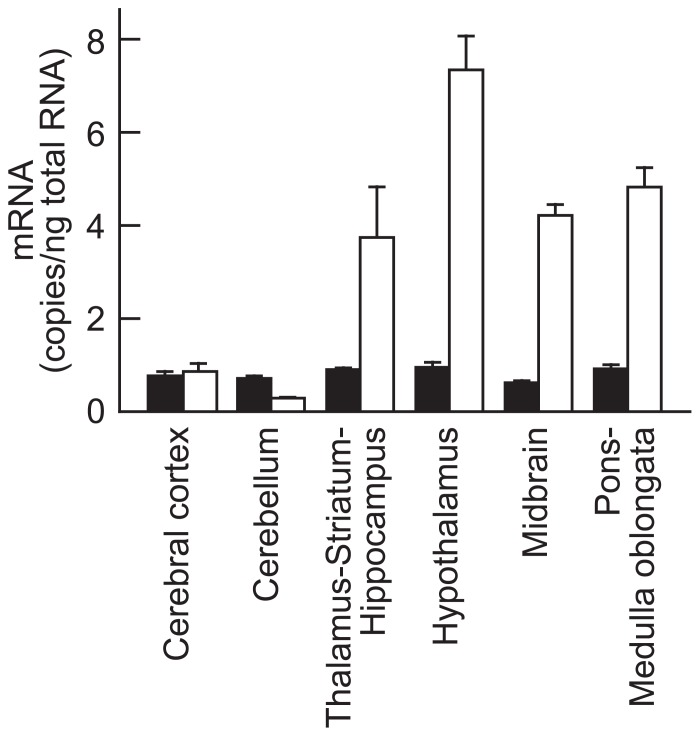
**Expression of neuromedin U receptors types 1 (NMUR1) and 2 (NMUR2) mRNA in rat brain**. Rat NMUR1 (closed bars) and NMUR2 (open bars) mRNA were quantified by quantitative reverse transcription–polymerase chain reaction analysis. Data represent means ± SEM of three rats.

To elucidate the regional distribution of NMS receptor in rat brain, the mRNA expression level of NMUR1 and NMUR2 was analyzed by quantitative PCR (**Figure 5**). NMUR1 mRNA was widely distributed throughout the brain with low level of expression (approximately 0.8 copies/ng total RNA). On the other hand, abundant expression of NMUR2 mRNA was observed not only in the hypothalamus (7.27 ± 0.81 copies/ng total RNA, mean ± SEM), but also in the thalamus–striatum–hippocampus (3.72 ± 1.11 copies/ng total RNA), midbrain (4.20 ± 0.16 copies/ng total RNA), and pons–medulla oblongata (4.76 ± 0.40 copies/ng total RNA).

### LEVELS OF NMS PEPTIDE IN THE BRAIN OF RATS MAINTAINED UNDER LIGHT/DARK CYCLING, AND OF FASTED RATS

**FIGURE 6:**
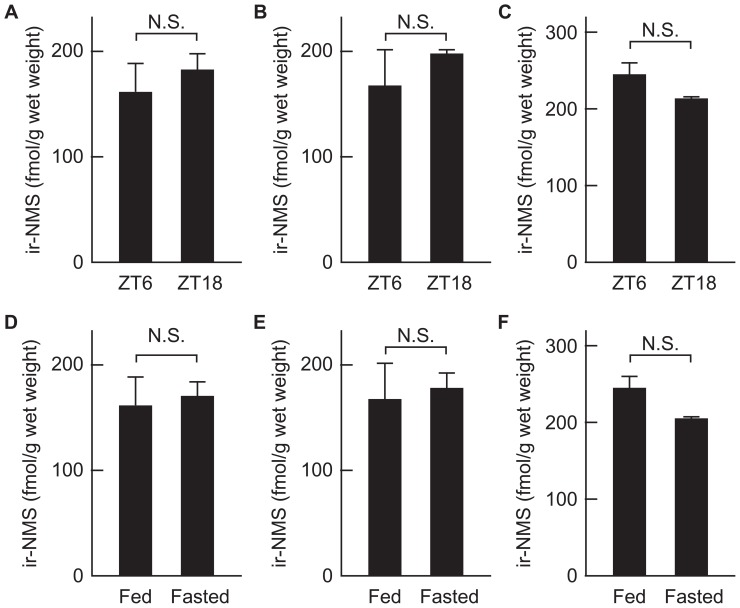
**Levels of immunoreactive neuromedin S (ir-NMS) in the brain of rats maintained under light/dark cycling, and of fasted rats**. **(A–C)** Concentrations of ir-NMS were quantified in the hypothalamus **(A)**, midbrain **(B)**, and pons–medulla oblongata **(C)** of rats under the light at ZT6 and dark at ZT18. **(D–F)** Concentrations of ir-NMS were quantified in the hypothalamus **(D)**, midbrain **(E)**, and pons–medulla oblongata **(F)** of free-fed and 48 h-fasted rats. Data represent means ± SEM of three rats. Data were analyzed statistically by Student’s *t*-test.

Most abundant expression of NMS mRNA is found in the SCN within the brain, and its expression level fluctuates under light/dark cycling (Mori et al., 2005). Therefore, we measured the levels of NMS peptide in the hypothalamus, midbrain, and pons–medulla oblongata of rats during both light and dark periods. The brains were sampled during the light period at ZT6 and the dark period at ZT18. In these brain regions, there is no significant difference in peptide levels between groups of rats at ZT6 and ZT18 (**Figures 6A–C**).

On the other hand, because ICV-administered NMS suppresses the food intake (Ida et al., 2005), we examined the effect of fasting on the levels of NMS peptide in the hypothalamus, midbrain, and pons–medulla oblongata. The peptide levels in the brain regions tested did not significantly differ between groups of free-fed and 48-h fasted rats (**Figures 6D–F**).

## DISCUSSION

We developed a highly sensitive RIA for rat NMS; the lowest detection limit was 0.25 fmol/tube (equivalent to 10.6 pg/ml). We evaluated the specificity of the assay by using a peptide extract of rat whole brain, because NMS was originally isolated from this tissue (Mori et al., 2005). The dilution curve for rat brain extract paralleled the standard curve. Moreover, no immunoreactivity other than that originating from native (major) and oxidized (minor) NMS was detected in any of the fractions of extracts separated by RP-HPLC, suggesting that the OB1321-2 antiserum is not cross-reactive to other neuropeptides that are abundantly expressed in the brain. This is no doubt because NMS shows no sequence homology with any other known neuropeptide except for NMU, a structurally related peptide. In addition, this RIA is unable to detect rat NMU. These data indicate that the assay was highly specific.

Within the rat brain, the distribution profile of ir-NMS was different from that of NMS mRNA. It was interpreted that we clearly detected NMS peptide in the hypothalamus, because its mRNA was abundantly expressed in this region. However, we also detected NMS peptide in the midbrain and pons–medulla oblongata at levels comparable to those in the hypothalamus, whereas the mRNA levels in these regions were much lower than in the hypothalamus. Differences in the distributions of the orexin peptide and its mRNA have also been observed. Expression of orexin mRNA is restricted to the lateral hypothalamic area (LHA) and posterior hypothalamus (PH; Sakurai et al., 1998), whereas orexin peptides are distributed in various areas of the brain (Mondal et al., 1999). Furthermore, immunohistochemical mapping has shown that orexin-containing nerve fibers are abundant in various brain areas, including the olfactory bulb, cerebral cortex, thalamus, hypothalamus, and brainstem (Date et al., 1999). These data indicate that orexin neurons in the LHA or PH send projections widely across the brain (Date et al., 1999). The predominant expression of NMS mRNA only in the hypothalamus thus indicates that the cell bodies of the NMS neurons are located mainly in this region. In addition, NMS-containing nerve fibers are likely to be present in the midbrain and pons–medulla oblongata, because NMS peptide was abundant in these regions despite the low levels of expression of NMS mRNA. On the other hand, it is likely that there are fewer NMS-producing cells in the spinal cord than in the hypothalamus, because NMS mRNA expression levels are low in the spinal cord, as they are in the brainstem (Mori et al., 2005). Therefore, it is likely that nerve fibers originating from hypothalamic NMS neurons project to the brainstem, which consists of midbrain, pons, and medulla oblongata.

In our previous study, quantitative RT-PCR and *in situ* hybridization analyses showed that NMS mRNA was predominantly expressed in the SCN of the hypothalamus (Mori et al., 2005), indicating that, within the hypothalamus, most NMS neurons are located in this nucleus. Together with this situation, in view of the larger distribution in the brain of NMS peptide compared to NMS mRNA, we speculated the existence of neural circuits from NMS neurons located in the SCN to the brainstem. Little is known that the nerve fibers originating from SCN neurons project directly to the brainstem, although it is reported that the SCN indirectly projects to the locus coeruleus of pons (Aston-Jones et al., 2001) and the ventral tegmental area of midbrain (Luo and Aston-Jones, 2009). The results we described here may lead to the discovery of novel neural circuits. On the other hand, another possibility is that NMS peptide in the brainstem comes from hypothalamic nuclei other than the SCN, such as the PVN that are known to project to various targets in the brainstem (Geerling et al., 2010), whereas the levels of NMS mRNA in the PVN and other hypothalamic nuclei are extremely low compared with that in the SCN (Mori et al., 2005). To clarify these possibilities, immunohistochemical analysis of NMS in the brain is needed, and further investigation is continued in this regard.

There are two kinds of receptors for NMS, NMUR1 and NMUR2 (Brighton et al., 2004; Mori et al., 2005), and their expression patterns are unique. Expression of NMUR1 is widely distributed throughout various tissues, and high levels of expression are found in peripheral tissues (Fujii et al., 2000). In contrast, NMUR2 mRNA is mainly expressed in the central nervous system (Hosoya et al., 2000), and its expression is clearly detected in the PVN, the wall of the third ventricle in the hypothalamus, and the CA1 region of the hippocampus (Howard et al., 2000). In addition, regional distribution of these receptors in rat brain was investigated in this study. Quantitative RT-PCR showed the apparent expression of NMUR2 mRNA in the midbrain and pons–medulla oblongata as well as in the hypothalamus. These data suggest the functional possibility of NMS within the brainstem.

We and another group have demonstrated that ICV administration of NMS induces a phase shift in the circadian rhythm (Mori et al., 2005), suppression of food intake (Ida et al., 2005), reduction of urine volume (Sakamoto et al., 2007), increased in milk ejection (Sakamoto et al., 2008), and release of luteinizing hormone (Vigo et al., 2007). All of actions are induced as a result of the direct effects of NMS on hypothalamic nuclei such as the SCN, ARC, PVN, and SON (Ida et al., 2005; Mori et al., 2005; Sakamoto et al., 2007, 2008; Vigo et al., 2007). On the other hand, NMS peptide and NMUR2 mRNA are present in the brainstem, suggesting the physiological role of NMS system in this region. However, the function of NMS by direct action on the brainstem remains to be clarified. The brainstem is important for basic bodily functions (especially regulation of the cardiovascular and respiratory systems). Therefore, regulation of these systems may be a major role of NMS within the brainstem, although it is not yet certain whether this action is a result of a direct effect of NMS in this region. On the other hand, our group recently reported that, in mice, NMS regulates the heart rate through the sympathetic nervous system (Sakamoto et al., 2011).

In the rat brain, NMS mRNA is predominantly expressed in the SCN, with weak expression in other nuclei of hypothalamus, and its expression fluctuates within the SCN under light/dark cycling: the largest decrease occurs during the dark period, followed by a gradual increase until the late light period (Mori et al., 2005). In contrast, there is no difference in hypothalamic content of NMS peptide between groups of rats during the light and dark periods. In general, the neuropeptide is stored in the secretory vesicles of neuron, and released in response to relevant stimuli. In view of this mechanism, discrepancy between mRNA and peptide levels of NMS may be caused by mechanism of storage and release of the neuropeptide as well as differences in mRNA and peptide turnover rates, because tissue content of peptide is modulated by the presence or absence of releasing stimuli, even if the expression level of mRNA is constant. On the other hand, hypothalamic content of NMS peptide was not influenced by fasting, although NMS is an anorexigenic peptide (Ida et al., 2005). To consider the relationship between hypothalamic content of peptide and physiology of NMS, further investigation is needed.

The negligible detection of NMS peptide in the spleen and testis was unexpected, because NMS gene expression levels in these tissues are higher than that in the whole brain (Mori et al., 2005). Several neuropeptides are produced in the spleen and testis. For example, somatostatin and its mRNA are detected in the spleen and pituitary adenylate cyclase-activating polypeptide and its mRNA are detected in the testis, indicating that both neuropeptides are synthesized and stored until release in these tissues (Aquila et al., 1991; Shioda et al., 1994). In contrast, because only NMS mRNA – not its peptide – was detected in the spleen and testis, NMS peptide accumulation is likely to be difficult in these tissues because NMS may be secreted *via* constitutive secretory pathways in the spleen and testis, similar to the way in which adrenomedullin is secreted from both endothelial and vascular smooth muscle cells (Sugo et al., 1994a, b). This may occur because the sequences of the cleavage sites involved in post-translational modification of the NMS precursor coincide with those of the consensus motifs (Arg-X-Lys/Arg-Arg and Arg-X-Arg-X-X-Arg) for proteolytic processing by furin and/or furin-like proteases (proprotein convertases functioning within constitutive secretory pathways; Seidah and Chrétien, 1999; Mori et al., 2005). On the other hand, there is another possible explanation as follows: if NMS mRNA is highly expressed in a discrete cell population within the spleen and testis, the peptide gets massively diluted when whole organ is homogenized. To verify these hypotheses, it is important to identify the NMS-producing cells in the spleen and testis by *in situ* hybridization analysis and immunohistochemistry.

In conclusion, we developed a highly sensitive and highly specific RIA for rat NMS. The different distribution of NMS peptide and its mRNA within the brain suggested the direct projection of nerve fibers originating from hypothalamic NMS neurons to the brainstem. In addition, the gene expression of NMS receptor, NMUR2, was clearly detected in the brainstem. Because the functions of NMS within the brainstem have not yet been identified, our findings provide a foundation on which further research can build to provide novel insights into the physiological roles of NMS in the brain.

## Conflict of Interest Statement

The authors declare that the research was conducted in the absence of any commercial or financial relationships that could be construed as a potential conflict of interest.
